# Changing Income-Related Inequality in Daily Nutrients Intake: A Longitudinal Analysis from China

**DOI:** 10.3390/ijerph17207627

**Published:** 2020-10-19

**Authors:** Yongjian Xu, Siyu Zhu, Yiting Zhou, Andi Pramono, Zhongliang Zhou

**Affiliations:** 1 School of Public Policy and Administration, Xi’an Jiaotong University, Xi’an 710049, China; zhusiyuxjtu@126.com (S.Z.); zzyytttt@163.com (Y.Z.); zzliang1981@xjtu.edu.cn (Z.Z.); 2 Community and Aged Care Services, Hunter New England Health, Wallsend 2287, NSW, Australia; Andi.Pramono@health.nsw.gov.au

**Keywords:** income-related inequality, nutrients intake, concentration index, health-related income mobility

## Abstract

Because of economic reform, dietary pattern in China changed rapidly during the past two decades. Meanwhile, the changes of income and nutrients intake had the same trend. This study aims to measure the income-related inequality in daily nutrients intake and its health-related income mobility over time. Data was sourced from four waves of China Health and Nutrition Survey. Concentration indexes and health-related income mobility indexes were employed to measure the income-related inequality of nutrients intake and its change over time. This study found that the daily protein intake, daily fat intake, daily energy intake, and proportion of energy from fat over 30% were more concentrated on the rich, whereas daily carbohydrates intake among the poor. The income-related inequalities were more severe than the cross-sectional perspective in the long run. The dynamic change of urbanisation indexes has resulted that over 30% of energy from fat was more concentrated among the rich and carbohydrates intake among the poor. The nutrition transition may bring about more severe disease economic burden to the poor in the future. This study recommends an approach to minimize gaps between rural and city areas by promoting rural revitalization to reduce the income-related inequality in daily nutrient intake.

## 1. Introduction

Nowadays, China is a country facing the nutrition “double burden” as a result of having similar proportions of people/community being both obesity and underweight. Data from the World Health Organisation (WHO) showed that in 2016 while 4.9% of people were underweight (body mass index (BMI; kg/m^2^) ≤ 18.5), 33.8% of people were overweight (BMI ≥ 25), and 6.6% were obese (BMI ≥ 30) [1]. Although the prevalence of obesity in China is still lower than that in Economic Co-operation and Development (OECD) countries, there has been an alarming increase in obesity in the past decades. The prevalence of obesity in 2016 was 11 times more compared with that in 1980 which was just 0.6% for Chinese adults. Due to the fact that many chronic noncommunicable diseases are often associated with obesity (e.g., type 2 diabetes), the dramatic increase in obesity may generate substantial economic burden to society at individual, community, and national levels. Among all the causes, what kinds of food people take plays an important role in developing obesity [2]. If energy intake is greater than energy output, body weight will increase, and vice versa [3].

With the economic reform and its opening to the outside world, China has experienced unprecedented economic growth and structural change in past decades [4]. In accordance with the development of social economy, dietary pattern in China has dramatically shifted from consuming high-carbohydrates and high-fiber foods to consuming high-fat and high-energy-density foods [5]. Unfortunately, the diet quality has not improved with the increase of Chinese national economy and urbanisation.

Previous studies focused mostly on tracking the change of dietary pattern over years as well as the effect of nutrients intake on health for Chinese population [2,6,7]. These studies have found that the quality of Chinese diet had improved in the past years. Moreover, different dietary patterns have been found to be associated with different health conditions [8,9,10,11,12]. The modern pattern (high loadings of with fast food, fried products, cakes, and milk) was positively associated with the growing prevalence of cardio metabolic risks, overweight and obesity, insulin resistance, and hypertension. Conversely, the traditional pattern (high loadings of with rice, vegetables, and meat) was persisting and it was considered to be a protective factor from cardio metabolic risks including overweight and obesity. So far, none of the studies have been published to investigate the distribution of nutrients intake between income groups. Since nutrients intake of the poor could pose more severe health hazards, and consequently result in more serious harm to the whole society, it is necessary to study the income-related inequality among the Chinese. Our study has two objectives. Firstly, to measure the income-related inequality in different kinds of food nutrients and its changes over seven years period. Secondly, to further study what caused the mobility of income-related inequality in nutrients intake. Our study could provide readers with a clearer picture on the severity of inequality in nutrients intake and what contributed this mobility in the long run. The results could also shed lights on policy formulation on how to reduce the nutrients intake inequality in other developing countries.

## 2. Materials and Methods 

### 2.1. Study Design

This is a retrospective cohort study designed to investigate the income-related inequality in nutrients intake over seven years.

### 2.2. Data

This study used the data from four waves (2004, 2006, 2009, 2011) of China Health and Nutrition Survey (CHNS). The CHNS is an ongoing cohort survey supported by the Carolina Population Center at the University of North Carolina at Chapel Hill and the National Institute of Nutrition and Food Safety at the Chinese Center for Disease Control and Prevention. The first wave of CHNS was conducted in 1989, and subsequent rounds were taken place every two to four years. A multistage, random cluster process was utilized to draw the sample in each wave. 

CHNS collects the information on residents’ diet structures, nutritional and health status, as well as socioeconomic conditions. Detailed information on quality assurance measures and sampling procedure could be found on their website [13].

Our study only focused on adults aged 18 years old and over with the use of four rounds of CHNS survey data collected in 2004, 2006, 2009, and 2011. Adults with complete information on each wave were included in the study, indicating balanced panel design was adopted in the study. In total, 4274 adults in baseline were included in our study, collecting 17,096 responses through 4 survey years. 

## 3. Measures

### 3.1. Nutrients Intake 

Individual’s food consumption (24 h recall) for three consecutive days were recorded by trained interviewers through face-to-face interviews in CHNS. All kinds of food consumed at home or away from home were all recorded. To minimize the recall bias of food eaten during the previous days, food models and pictures were used to aid the respondents in recalling the consumed food types, and the amounts. China food composition table, which presents nutrient components for each food item, was used to convert the food consumption into nutrients intake [14,15,16]. The daily intake of protein, fat, and carbohydrates for each respondent were calculated by summing up nutrients intake in the three consecutive days and dividing it by three. Daily energy intake (kJ) was then calculated on the basis of consumed quantity (gram) recorded in the dietary recall data. Since each gram of fat supplies the body with about nine calories, the proportion of energy from fat was obtained by multiplying the number of grams of fat by nine and dividing by total energy intake. 

### 3.2. Other Variables

There are many different variables available in CHNS. Based on previous empirical studies, potential variables that may be associated with macronutrients intake were initially considered in the study [17,18]. Sociodemographic characteristics considered in this study included age, gender, education (illiterate, racy, elementary, middle school, high school, and university), marital status (unmarried, married, others), geographic regions (Eastern China, Central China, and Western China), working status, and urbanization index. Urbanization index, which was developed by Jones-Smith and Popkin, is a comprehensive composite variable to measure the urbanization level of each communities/villages through 12 aspects of urbanization: population density, communication, modern markets, sanitation, social services, et al. [19,20]. Economic status was obtained by equally grouping individuals into the poorest, poorer, middle, richer, and the richest quintiles according to per capita net household expenditure, that is, total household expenditure in the last year minus health expenditure, and divided by the household size. Two dietary-related variables were also included as covariates. Dietary knowledge was a continuous variable that was constructed from 12 questions preselected in CHNS. 5-point Likert scale was used in each question: “strongly disagree”, “disagree”, “neutral”, “agree” and “strongly agree”. For each negative question, we assigned a score ranging from 0–2, with 2 for strongly disagree, 1 for disagree, and 0 for other answers, and vice versa. The total dietary knowledge score lies between 0–24. Questions on five kinds of foods were preselected in the CHNS to investigate individual’s preference on fast foods, salty snack foods, fruit, vegetables and soft drinks/sugary fruit drinks. Five-point Likert scale (dislike very much, dislike, neutral, like, like very much) was used to measure the degree of their preference. A score ranging from 1–5 for each category was generated to measure the preference degree with a higher score indicating a healthier preference. A summary of food preference score was then generated to measure the overall degree of healthy preferences with the sum of scores on five questions. The total score for food preference lies between five and 25.

### 3.3. Statistical Analyses

The means and standard errors were employed to describe continuous variables. The percentages were used to describe categorical variables. Over the past thirty years, as an example of a hierarchical structure with repeated observations over time nested within individuals, multilevel model has become a widely used approach in the analysis of longitudinal data. Multilevel models can be applied when events are repeatable to allow for correlation between the durations to events experienced by the same individual, or when individuals are clustered into higher-level units [21]. In multilevel models for longitudinal data, repeated measures have a two-level hierarchical structure with measurements at level 1 nested within individuals at level 2. The empty model without any independent variables (random intercept null model) are presented as follows:

Level 1 repeated-measures level model:

(1) $$y_{ti}=\beta_{oi}+e_{ti}$$

where *t* is the measurement occasions at time *t*. i represents the ith individuals. $y_{ti}$ is the estimated average score of dependent variables for ith individuals at time *t*, *e_ti_* is the error term which captures the within-individual variation.

Level-2 individual level models:

(2) $$\beta_{oi}=\gamma_{00}+U_{0i}$$

$\gamma_{00}$ is the grand mean of the dependent variable, $U_{0i}$ is the difference between the *i*th average dependent variable score and the grand mean, *U* is assumed to be normally distributed with the expected value of 0 and the variance $\sigma_{u}^{2}$.

The multi-level random intercept model with independent variables can be written as follows:

(3) $$y_{ti}=\beta_{oi}+\beta_{1}{}_{i}x_{1}{}_{ti}+e_{ti,}$$

where $\beta_{1}{}_{i}$ is unknown coefficients to be estimated. 

Concentration index is a leading approach used to measure socioeconomic health inequalities in health economics. Due to the illustrative and interpretation, the concentration index enjoys an increasing popularity as an indicator of the inequality of health in relation to the socioeconomic position of individuals. 

The cross-sectional concentration index $C^{t}$ was firstly introduced by Kakwani and Wagstaff et al. [22]. It is defined as follows:

(4) $$C^{t}=\frac{2}{{\bar{h}}^{t}}cov\left(h_{it},R_{i}^{t}\right)=\frac{2}{N{\bar{h}}^{t}}\sum^{\;}\left(h_{it}-{\bar{h}}^{t}\right)\left(R_{i}^{t}-\frac{1}{2}\right);t=1,\ldots T,\;\;\;\;\;\;\;\;\;$$

where $C^{t}$ denotes the cross-sectional concentration index of health at time t, $h_{it}$ is the mean health of individual *i* in period *t*, $R_{i}^{t}$ is relative rank of individual i in the distribution of *N* incomes in period *t*. 

It is a measure of the socioeconomic inequality in health based upon information on the socioeconomic ranks and the health levels of all individuals in the population. A positive index indicates that health is more concentrated in the rich, whereas the negative index indicates that health is more distributed in the poor [23]. Therefore, if the health variable is expressed positively among the rich, a positive index suggests a pro-rich distribution, and vice versa. The higher the absolute value of the concentration index, the more extreme the pro-rich or pro-poor character of the distribution of health is. Cross-sectional concentration index, however, cannot capture the positive association between income and health variable that respondents may experience over time. A long-run concentration index was then introduced to capture this association in the measurement of inequality over time. The longitudinal concentration index can be written as follows:

(5) $$CI^{T}=\frac{2}{{\bar{h}}^{T}}cov(h_{i}^{T}h_{i}^{T},R_{i}^{T})=\frac{2}{N{\bar{h}}^{T}}\underset{i}{\sum }\left(h_{i}^{T}-{\bar{h}}^{T}\right)\left(R_{i}^{T}-\frac{1}{2}\right)$$

Jones showed that the longitudinal concentration index could be written as the subtraction of two terms [24,25]. Term 1 is the weighted sum of cross-sectional concentration indexes for each period. Term 2 is the difference between period specific income ranks and ranks for mean income over all time periods and their relationship to health.

(6) $$CI^{T}=\underset{t}{\sum }w_{t}CI^{t}-\frac{2\sum_{i}\sum_{t}\left(h_{it}-{\bar{h}}^{t}\right)\left(R_{i}^{t}-R_{i}^{T}\right)}{NT{\overset{=}{h}}^{T}}\;=\underset{t}{\sum }w_{t}CI^{t}-\frac{2\sum_{i}\sum_{t}\left(h_{it}-{\overset{=}{h}}^{T}\right)\left(R_{i}^{t}-R_{i}^{T}\right)}{NT{\overset{=}{h}}^{T}}$$

Modelled on Shorrocks’s index of income mobility, Jones defined an index of health-related income mobility that measures how much long-run perspectives alters the picture that would emerge from a series of cross-sections.

(7) $$M^{T}=\frac{\sum_{i}w_{t}CI^{t}-CI^{T}}{\sum_{i}w_{t}CI^{t}}=\frac{\left(\left(2/NT{\overset{=}{h}}^{T}\right)\sum_{i}\sum_{t}\left(h_{it}-{\overset{=}{h}}^{T}\right)\left(R_{i}^{t}-R_{i}^{T}\right)\right)}{\sum_{i}w_{t}CI^{t}}$$

Jones further showed that the index of health-related income mobility can be decomposed into the contributions of determinants based on econometric model.

(8) $$M^{T}=\overset{k}{\underset{k=1}{\sum }}{\hat{\beta }}_{k}\frac{\sum_{t}{\bar{x}}_{k}{}^{t}CI^{t}{}_{xk}}{\sum_{t}{\bar{y}}^{t}CI^{t}}M^{T}{}_{xk}+\varepsilon$$

where ${\hat{\beta }}_{k}$ is the estimated coefficients from regression model, ${\bar{x}}_{k}{}^{t}$ and ${\bar{y}}^{t}$ are the mean of independent and dependent variables, respectively, $CI^{t}{}_{xk}$ and $CI^{t}$ are cross-sectional concentration indexes for independent variables and dependent variables, respectively.$\;{\hat{\beta }}_{k}\frac{\sum_{t}{\bar{x}}_{k}{}^{t}CI^{t}{}_{xk}}{\sum_{t}{\bar{y}}^{t}CI^{t}}$ is the elasticity of dependent variable with respect to independent variables. ε is the residual.

All statistical analysis was computed using the SAS (SAS Institute, Cary, NC, USA) and Stata (StataCorp, College Station, TX, USA) software.

## 4. Results

Table 1 shows characteristics of respondents at baseline. The mean (SD) age of the study group was 49.19 (12.68%) years old with 1966 (46%) men and 2308 (54%) women. Among 4272 respondents, 3836 (89.75%) of them were married, just 149 (3.49%) had the education attainment of university, and 2006 (46.93%) lived in Eastern China.

**Figure 1:**
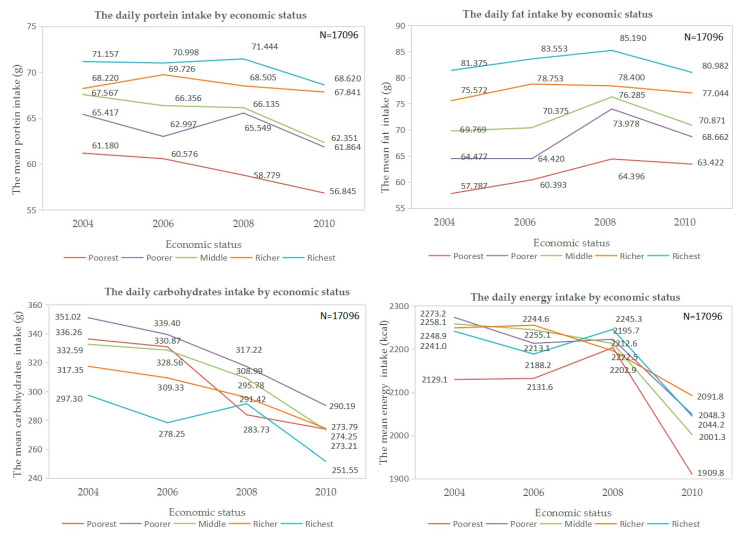
The daily nutrients intake of protein, fat, energy, and carbohydrates by economic groups.

Figure 1 shows the daily nutrients intake of protein, fat, energy, and carbohydrates by economic groups. From 2004–2011, the richest consistently had the highest, and the poorest had the lowest daily protein and fat intake, respectively. Although the daily energy intake overlapped among the richest, richer, middle, and poorer, it was evident that the poorest had the lowest energy intake among all economic groups. The richest had the lowest daily carbohydrates intake among all income groups.

Table 2 shows the proportions of Chinese adults having more than 30% energy from fat over time by economic groups. The poorest had the fastest increase in proportions of having more than 30% energy from fat, jumping from 26.11–58.41%. Although the richest had the highest proportions of having more than 30% energy from fat in each year, the proportion of the increase was the least among all income groups from 2004–2011.

Table 3 shows the association between nutrients intake and its determinants among Chinese adults.

Results from two level multiple linear regression random intercept model showed that age was negatively associated with daily protein, fat, energy, and carbohydrates intake. Conversely, income was positively associated with daily protein, fat, energy, carbohydrates intake, and the proportion of energy from fat over 30%. The daily intake of protein, fat, energy, and carbohydrates for women were significantly lower than that in men. The effect of marital status on daily nutrients was heterogeneous. 

While there was statistical significance of the associations between marital status and daily fat intake and energy intake, the statistical associations between marital status and protein and carbohydrates intake were not observed. Dietary knowledge was positively associated with daily fat intake, but negatively associated with daily energy and carbohydrates intake. Urbanization index was positively associated with daily protein intake, daily fat intake, proportion of energy from fat over 30%, but negatively associated with energy and carbohydrates intake.

Table 4 shows the concentration and mobility indexes for daily protein intake, daily fat intake, daily energy intake, daily carbohydrates intake, and proportion of energy from fat over 30%, respectively. The cross-sectional and longitudinal concentration indexes of nutrients intake on income in each wave of CHNS were presented in CI^t^ and CI^T^ column, respectively. These cross-sectional and longitudinal concentration indexes for daily protein intake, daily fat intake, daily energy intake, and proportion of energy from fat over 30% were all positive, indicating that these nutrients intake were more concentrated on the rich regardless in the short or long run. The cross-sectional and longitudinal concentration indexes of daily carbohydrates intake were consistently negative, indicating that the carbohydrates intake was more concentrated on the poor in the short and long run. Term 1 presents the weighted average of the cross-sectional concentration indexes up to the corresponding wave. There was a slight upward trend in terms of daily protein intake, as the cross-sectional concentration indexes for the middle and later periods were larger than the baseline. There were slight downward trends in term of daily fat intake, carbohydrates intake, and proportion of energy from fat over 30%, since the inequalities in later periods were smaller than the baseline. The decrease in inequality in nutrients intake within some of the periods contributed to the reduction. The term 2 captures the difference between income ranks of specific period and ranks for average income over all periods and their relationship to nutrients intake. The term 2 for daily protein intake, daily fat intake, proportion of energy from fat over 30%, and daily energy intake were all negative. This made long-run income-related inequality greater than what we could infer from the cross-sectional measures. This effect increased the long-run income-related health inequality for daily protein intake, fat intake, proportion of energy from fat over 30%, and energy intake (8.2%, 19.7%, 12.4%, and 24.6%, respectively). These increases were reflected by the mobility indexes of these nutrients’ intake. Since the weighted average concentration index for daily carbohydrates intake was negative, and the term 2 was positive after four waves, the longitudinal concentration index for daily carbohydrates intake was larger than cross-sectional concentration index by 9.8%.

Table 5 presents the contribution of each determinants to the health-related income mobility indexes after four waves. The second column shows the longitudinal concentration indexes of independent variables. A positive longitudinal concentration index indicates that the variable is more concentrated in the rich and a negative indicates that this variable is more concentrated in the poor. Higher education, being married, household with 2–5 members, higher dietary knowledge, and higher urbanization index were more concentrated among the rich, whereas living in Central and Western China were more concentrated among the poor in the long-run perspective. Mobility indexes of independent variables are shown in the third column. A negative mobility index implies that the weighted average of short-run indexes underestimates the degree of long-run inequality (whether pro-rich or pro-poor), and vice versa. The mobility index of −0.6317 for age indicated that the short-run measure underestimated long-run pro-rich inequality by 63.17%. The mobility index of −0.2298 for married indicated that the short-run measure underestimated long-run pro-rich inequality by 22.98%. The mobility index of −1.6982 for middle school indicated that while the cross-sectional measures would suggest the inequality concentrated on the poor, there was an actual inequality that concentrated in the rich in the long run.

The contribution column shows the actual contribution of each independent variable to the index of health-related income mobility. The dynamic of richest income groups, ceteris paribus, reduced the degree of inequality for daily protein, fat, energy, carbohydrates intake, and proportion of energy from fat over 30% that concentrated in the rich in the long run. The richest income groups contributed the inequality increased of 14.86% for daily energy intake, and 25.12% for proportion of energy from fat over 30%. Urbanization index increased carbohydrates intake more concentrated among the poor 10.25% in the long run.

## 5. Discussion

Macronutrients are nutrients that the body uses and needs daily in relatively large amounts. There are three macronutrients: proteins, carbohydrates, and fats. Macronutrients provide individual’s body with calories (energy) and building blocks for cellular growth, immune function, and overall repair. Previous studies have investigated the trend of daily nutrition intake [26]. However, the differences of nutrients intake between the advantaged and the disadvantaged were less reported. Based on the analysis of nutrients intake for adults from 2004–2011, our studies have several findings. Our study firstly observed macronutrients intake trend for Chinese adults from 2004–2011. A clear downward trend in daily protein intake for the poorest was showed from 2004–2011. Protein is a vital substance in the maintenance of body tissues, including development and repair. Most of human body and organs are all made from protein. Protein also acts as a major source of energy, a major element in transportation of certain molecules, and forms antibodies that help prevent infection, illness and disease. However, overconsumption of protein could also bring negative effects on health. Although the protein intake of 68 g per person per day for the Chinese adults was lower than average protein consumption in the world, the intake is above the recommended daily allowance which is 46 g for women and 56 g for men per day [27]. Public policies and intervention measures can be initiated to encourage people to consume the right amount and the right kinds of protein to get its health benefits. Carbohydrates are the main source of energy. However, consuming too many or the wrong types of carbohydrates might lead to health problems, e.g., feeling tired, obesity, gastrointestinal distress. As impact of the remarkable economic growth and rapid urbanisation, industrial food with nice flavouring but contains higher carbohydrates has gradually replaced traditional diet. Fortunately, in line with most previous studies, our study showed that the daily carbohydrates intake decreased dramatically from 2004–2011 [26,28,29]. In the meantime, the total energy intake also declined from 2004–2011. This may be attributed to the life-style changes over the preceding decades. Previous observational studies showed that there had been a trend towards an increase in sedentary lifestyles in the past decades [30]. Data from WHO showed that the crude obesity prevalence remarkably increased from 3% in 2004 to 4.8% in 2011, however, the daily energy intake decreased from 2230.07–2019.24 kcal at the same time [31]. Obesity is widely considered to be a result of either excessive energy intake or of insufficient physical activity. However, it is still controversial whether the excessive energy intake or lack of physical activity deserves the major responsibility for obesity. Previous studies have shown that reducing obesity should modify both energy intake and energy consumption, rather than simply focusing on either alone [32]. Therefore, in today’s sedentary environment, although the energy intake is steadily declining, the obesity prevalence will still increase. Matching daily energy intake to energy expenditure may be a feasible way for most people to maintain a healthy body. Our study observed that the daily fat intake and the proportion of energy from fat over 30% overall showed an increasing trend from 2004–2011. This trend was also observed in many developed and developing countries in the world [33]. With economic progress and globalisation, industrialized foods have become more available, which is tastier but with more added fat than the traditional foods. Over consumption of these type of foods might consequently make people gain weight, become fatter and unhealthier. 

Our study investigated the distribution of nutrients intake among different economic status groups. Results showed that there was a difference in daily protein intake between the rich and the poor in each year. In addition, the gap of daily protein intake quantities between the poor and the rich became wider from 2004–2011. This phenomenon was captured by relatively concentration indexes, which increased from 0.029–0.037. The rich group has higher ability to pay for foods with higher protein content. Our study found that there was income-related inequality in fat intake, with the richest having the highest quantity of daily fat intake and highest proportion of energy from fat. In the long-run perspective, this inequality was more severe in terms of cross-sectional view. However, it was worth noted that the poorest had the highest increase rate among all economic status groups from 2004–2011. Since the excessive fat intake is a predictor of numerous chronic diseases such as diabetes and stroke, this nutrition transition may lead to heavier economic burden to the poor in the future. The rapid increase in proportion of energy from fat among the poor may be something that we need to pay more attention to [34]. Unlike other macronutrient intake, our study found that the richest had the lowest carbohydrates intake compared to other income groups. Concentration indexes in each year also captured this phenomenon, and showed that the poor were more likely to have higher carbohydrates intake. This phenomenon was also observed in many developed countries [35,36]. The possible reasons are as follows: Firstly, the better off have richer dietary knowledge, so they are more likely to make rational choices on foods with lower carbohydrates, and stick to a healthy diet. Secondly, prices of healthy foods may also pose a significant barrier for the poor consumers trying to balance good nutrition with affordability. Since the richest had the highest total energy intake compared with other income groups, we can infer that the carbohydrates contributed less energy for the richest. Moderate carbohydrates intake can provide numerous health benefits. However, excessive amounts of carbohydrates intake can give negative impact on people’s health, longevity, and sustainability [37]. Moreover, dietary carbohydrates quality measured by dietary glycemic index, glycemic load, dietary fibre, and whole grains may play a more important role in population health than that amount [38,39,40]. Therefore, the difference in types and quality of carbohydrates intake between income groups should be put emphasis on further studies. Promoting changes in the dietary patterns of the poor may be an effective way to prevent economic burden due to chronic diseases in the future. Our study further decomposed health-related income mobility into two terms. Results showed that the term 2 of daily protein intake, fat intake, energy intake, and proportion of energy from fat over 30% in Table 4 were all negative after four waves. This indicated that individuals with lower income mobility were more likely to have below average intake level of these nutrients than individuals with higher income mobility. 

Our study further decomposed the index of health-related income mobility into its determinants. The study found that the richest group showed large contributions on health-related income mobility index of daily protein, fat, energy intake, and proportion of energy from fat over 30%. It is because this characteristic had a positive income mobility. Therefore, the degree of income-inequality concentrated in the rich would be reduced if a long-run average inequality perspective were taken. Besides, the elasticity of higher income was positive. Thus, the dynamic of income would ceteris paribus, decreased the degree of income-related inequality of daily protein, fat, energy intake, and proportion of energy from fat over 30% concentrated among the rich in the long run. This is the largest effect among the independent variables in these models. The uneven development between the rural and urban has become a long-term problem in China. In 2015, households in rural areas had 40 percent less disposable income than the urban households [41]. The higher urbanisation index was more concentrated among the rich in the long run, and it was positively associated with the proportion of energy from fat. Therefore, the proportion of energy from fat over 30% was more concentrated among the rich. Since the carbohydrates’ intake was more concentrated among the poor, and the association between carbohydrates intake and urbanization index were negative, the dynamic change of urbanisation index made daily carbohydrates intake more concentrated among the poor. Our study found age made the daily energy intake more concentrated among the rich, which is because the elderly was more concentrated among the poor using a long average inequality perspective, and the increase of age was negatively associated with the daily energy intake. 

Our findings implied that socioeconomic inequality in nutrients intake in Chinese adults was influenced not only by the functions of health system but also by factors beyond the scope of the health system. It highlights that future policies and intervention measures aim to reduce income-related inequalities in daily nutrients intake need to pay much more attention to the structure problems that trap adults in unhealthy nutrient intake rather than just focusing on transitory episodes of unhealthy nutrient intake. Therefore, implementing strategies that promote a balanced development between urban and rural areas could be a feasible way to reduce income-related inequality in nutrients intake.

There are some limitations in our study that need to be highlighted. Firstly, this is an observational study, therefore a causal conclusion cannot be drawn. Secondly, the identified determinants of the macronutrients intake in the study were all initiated from the pre-specified questions in the CHNS. Other unobserved confounders may have been omitted in multilevel models. Thirdly, all the information was self-reported, which may have resulted in a recall bias. Finally, study on diet quality especially in the quality of carbohydrates intake, protein intake, fat intake are not be available due to data access restrictions.

## 6. Conclusions

The daily intake of protein, energy, and carbohydrates were decreasing from 2004–2011, whereas the daily fat intake and the proportion of energy from fat over 30% showed an upward trend. There was income-related inequality in nutrients intake, with the rich having much more quantity of daily protein, fat, and energy intake, and the poor having much more carbohydrates intake. The relative income-related inequalities of daily protein intake had a tendency to become larger. It was noted that in the long run, the income-related inequalities were more severe than the cross-sectional perspective. The nutrition transition may bring about more severe disease economic burden to the poor in the future. Decomposition on the health-related income mobility showed that the dynamic of income would, ceteris paribus, decrease the degree of income-related inequality concentrated among the rich in the long run, while the dynamic of urbanisation index made the degree of income-related inequality in carbohydrates more concentrated among the poor and the proportion of energy from fat over 30% more concentrated among the rich. In addition, the difference in diet quality between income groups need to be studied on further studies. The results of this study imply that future policies and intervention measures that aim to reduce income-related inequalities in daily nutrients intake need to pay much more attention to social structure problems. The inequality in daily nutrient intake depends not only on health system functions but also on determinants beyond the scope of health authorities. This reveals that the Chinese government should pay more attention to socioeconomic factors. Our study suggests that eradicating barriers to promote rural revitalization and equal distribution of opportunities in rural areas may be beneficial to reduce the income-related inequality in daily nutrient intake. 

## Figures and Tables

**Table 1:** Characteristics of respondents at baseline.

Variables	All (*N* = 4274)
Age, years	
Mean ± SD	49.19 ± 12.68
Gender	
Men, *n* (%)	1966 (46)
Women, *n* (%)	2308 (54)
Income, RMB yuan	
Mean ± SD	5353.48 ± 5313.93
Education	
Illiterate, *n* (%)	524 (12.26)
Elementary, *n* (%)	1515 (35.45)
Middle school, *n* (%)	1339 (31.33)
High school, *n* (%)	747 (17.48)
University, *n* (%)	149 (3.49)
Marital status	
Unmarried, *n* (%)	152 (3.56)
Married, *n* (%)	3836 (89.75)
Others, *n* (%)	286 (6.69)
Household size	
1	96 (2.25)
2–5	3788 (88.63)
≥6	390 (9.12)
Dietary knowledge score	
Mean ± SD	3.67 ± 1.98
Working status	
No, *n* (%)	1565 (36.62)
Yes, *n* (%)	2709 (63.38)
Urbanization index	
Mean ± SD	60.31 ± 19.74
Geographic regions	2006 (46.93)
Eastern China, *n* (%)	2006 (46.93)
Central China, *n* (%)	1350 (31.59)
Western China, *n* (%)	918 (21.48)

**Table 2:** The proportions of Chinese adults having more than 30% energy from fat over time by economic groups.

Economic Status	2004		2006		2009		2011	
	N	%	N	%	N	%	N	%
Poorest	223	26.11	291	34.28	346	40.52	384	45.02
Poorer	269	31.50	308	35.81	404	47.31	378	44.16
Middle	331	38.76	372	43.56	438	51.17	437	51.35
Richer	415	48.48	453	53.23	496	58.08	503	58.56
Richest	500	58.41	550	63.95	554	64.72	560	65.50

**Table 3:** The association between nutrients intake and its determinants among Chinese adults.

Variables	Protein			Fat			Energy			Carbohydrate			Proportion of Energy from Fat over 30%		
	Estimate	Std. Error	*Ρ*	Estimate	Std. Error	*Ρ*	Estimate	Std. Error	*Ρ*	Estimate	Std. Error	*Ρ*	Estimate	Std. Error	*Ρ*
Age (years)	−0.2023	0.0203	<0.0001	−0.1784	0.0350	<0.0001	−7.2934	0.5937	<0.0001	−1.3183	0.0932	<0.0001	0.0037	0.0018	0.0403
Gender															
Men (ref)															
Women	−9.7073	0.4566	<0.0001	−8.9420	0.7811	<0.0001	−350.9100	13.4606	<0.0001	−45.2909	2.1302	<0.0001	0.1850	0.0401	<0.0001
Economic status															
Poorest(ref)															
Poorer	2.4282	0.5414	<0.0001	4.3307	0.9743	<0.0001	83.6144	15.5722	<0.0001	7.6141	2.3749	0.0013	0.1063	0.0543	0.0500
Middle	3.4332	0.5567	<0.0001	5.8832	0.9999	<0.0001	81.7213	16.0288	<0.0001	3.3460	2.4478	0.1717	0.2454	0.0550	<0.0001
Richer	5.0836	0.5820	<0.0001	8.3749	1.0422	<0.0001	106.7100	16.7764	<0.0001	3.1888	2.5666	0.2141	0.3908	0.0568	<0.0001
Richest	6.8708	0.6325	<0.0001	11.1779	1.1288	<0.0001	130.0900	18.2636	<0.0001	0.3954	2.8006	0.8877	0.5159	0.0613	<0.0001
Education															
Illiterate (ref)															
Elementary	−0.4242	0.6263	0.4982	2.9425	1.1006	0.0075	9.5151	18.2127	0.6014	−3.9324	2.8222	0.1635	0.1571	0.0591	0.0079
Middle school	0.5093	0.7121	0.4744	6.0396	1.2471	<0.0001	13.4468	20.7428	0.5168	−10.6401	3.2225	0.0010	0.3122	0.0664	<0.0001
High school	−0.2434	0.8218	0.7671	4.0749	1.4352	0.0045	−38.0573	23.9763	0.1125	−17.1813	3.7332	<.0001	0.3787	0.0760	<0.0001
University	−1.0356	1.2332	0.4011	1.1483	2.1538	0.5940	−86.3642	35.9761	0.0164	−20.7042	5.6014	0.0002	0.2803	0.1147	0.0146
Marital status															
Unmarried (ref)															
Married	1.7426	1.3882	0.2094	6.0781	2.4360	0.0126	104.6100	40.4034	0.0096	5.4535	6.2680	0.3843	0.0244	0.1313	0.8526
Others	−0.2356	1.6039	0.8832	7.2159	2.8102	0.0102	56.1322	46.7149	0.2295	−5.9605	7.2549	0.4113	0.2537	0.1504	0.0917
Household size															
1 (ref)															
2–5	1.8692	1.2715	0.1416	−13.1687	2.2415	<0.0001	−114.5000	36.9207	0.0019	3.2140	5.7083	0.5734	−0.4553	0.1223	0.0002
≥6	3.4912	1.3936	0.0123	−14.6821	2.4555	<0.0001	−117.1500	40.4780	0.0038	5.1180	6.2607	0.4137	−0.5975	0.1336	<0.0001
Dietary knowledge score	−0.0628	0.0472	0.1838	0.3277	0.0855	0.0001	−5.4799	1.3556	<0.0001	−2.0118	0.2061	<0.0001	0.0368	0.0048	<0.0001
Working status															
No (ref)															
Yes	3.3995	0.4306	<0.0001	2.7573	0.7679	0.0003	118.7900	12.4360	<0.0001	17.6471	1.9078	<0.0001	−0.1020	0.0416	0.0143
Urbanization index	0.0696	0.0125	<0.0001	0.2678	0.0216	<0.0001	−2.0606	0.3644	<0.0001	−1.2120	0.0570	<0.0001	0.0212	0.0011	<0.0001
Geographic regions															
Eastern China (ref)															
Central China	0.4276	0.4997	0.3921	0.3542	0.8529	0.6779	71.9742	14.7461	<0.0001	20.5747	2.3375	<0.0001	−0.1650	0.0437	0.0002
Western China	−4.7788	0.5789	<0.0001	−1.5162	0.9893	0.1254	−28.6851	17.0733	0.0930	4.7829	2.7041	0.0770	−0.0787	0.0505	0.1191

Note: The sample size is 17,096. Estimate: estimate of beta-coefficients.

**Table 4:** The concentration and mobility indexes for nutrients intake.

Variables	Year	CI^t^ (Std. Error)	Term 1	Term 2	CI^T^ (Std. Error)	M^T^
Protein	2004	0.029 (0.003)	0.029	0.000	0.029 (0.003)	0.000
	2006	0.035 (0.003)	0.032	−0.002	0.034 (0.003)	−0.069
	2009	0.036 (0.003)	0.033	−0.003	0.037 (0.002)	−0.098
	2011	0.037 (0.003)	0.034	−0.003	0.037 (0.002)	−0.082
Fat	2004	0.070 (0.004)	0.070	0.000	0.070 (0.004)	0.000
	2006	0.070 (0.004)	0.070	−0.009	0.079 (0.003)	−0.123
	2009	0.049 (0.005)	0.063	−0.010	0.073 (0.003)	−0.155
	2011	0.049 (0.006)	0.059	−0.012	0.071 (0.003)	−0.197
Energy	2004	0.008 (0.003)	0.008	0.000	0.008 (0.003)	0.000
	2006	0.006 (0.003)	0.007	−0.001	0.008 (0.002)	−0.139
	2009	0.018 (0.003)	0.011	−0.002	0.013 (0.002)	−0.196
	2011	0.012 (0.003)	0.011	−0.003	0.014 (0.002)	−0.246
Carbohydrate	2004	−0.028 (0.003)	−0.028	0.000	−0.028 (0.003)	0.000
	2006	−0.035 (0.003)	−0.031	0.003	−0.034 (0.003)	−0.099
	2009	−0.006 (0.003)	−0.023	0.002	−0.025 (0.002)	−0.093
	2011	−0.019 (0.003)	−0.022	0.002	−0.024 (0.002)	−0.098
Percentage energy from fat	2004	0.165 (0.010)	0.165	0.000	0.165 (0.010)	0.000
	2006	0.139 (0.010)	0.151	−0.019	0.170 (0.003)	−0.124
	2009	0.089 (0.008)	0.128	−0.016	0.143 (0.006)	−0.122
	2011	0.087 (0.008)	0.117	−0.014	0.131 (0.005)	−0.124

Note: the sample size is 17,096. CI^t^ is the cross-sectional concentration index; CI^T^ is the longitudinal concentration index.

**Table 5:** The contribution of each determinant to the health-related income mobility indexes.

Variables	CI(x) after 7 years	Mobility(x)	Elasticity					Contribution				
			Protein	Fat	Energy	Carbohydrate	Percentage Energy from Fat	Protein	Fat	Energy	Carbohydrate	Percentage Energy from Fat
Age (years)	−0.0114	−0.6317	0.0332	0.0154	0.1127	−0.0723	−0.0247	−0.0210	−0.0097	−0.0712	0.0456	0.0156
Gender												
Men (ref)												
Women	−0.0104	−0.1528	0.0602	0.0291	0.2047	−0.0937	−0.0460	−0.0092	−0.0044	−0.0313	0.0143	0.0070
Income												
Poorest (ref)												
Poorer	−0.3384	0.1557	−0.0868	−0.0812	−0.2811	0.0908	−0.1524	−0.0135	−0.0126	−0.0438	0.0141	−0.0237
Middle	−0.0509	−65.9785	−0.0002	−0.0002	−0.0005	0.0001	−0.0007	0.0153	0.0138	0.0343	−0.0050	0.0439
Richer	0.2739	0.3135	0.1807	0.1561	0.3568	−0.0378	0.5572	0.0567	0.0489	0.1119	−0.0119	0.1747
Richest	0.6635	0.1701	0.4904	0.4183	0.8733	−0.0094	1.4769	0.0834	0.0712	0.1486	−0.0016	0.2512
Education												
Illiterate (ref)												
Elementary	−0.1424	−0.1545	0.0077	−0.0279	−0.0162	−0.0237	−0.1139	−0.0012	0.0043	0.0025	0.0037	0.0176
Middle school	0.0301	−1.6982	0.0008	0.0049	0.0020	0.0055	0.0194	−0.0013	−0.0083	−0.0033	−0.0093	−0.0329
High school	0.3153	−0.1375	−0.0051	0.0450	−0.0755	0.1208	0.3202	0.0007	−0.0062	0.0104	−0.0166	−0.0440
University	0.6810	−0.0842	−0.0118	0.0068	−0.0922	0.0784	0.1277	0.0010	−0.0006	0.0078	−0.0066	−0.0107
Marital status												
Unmarried (ref)												
Married	0.0179	−0.2298	0.0100	0.0184	0.0567	−0.0105	0.0056	−0.0023	−0.0042	−0.0130	0.0024	−0.0013
Others	−0.1876	−0.2595	0.0014	−0.0223	−0.0311	−0.0117	−0.0600	−0.0004	0.0058	0.0081	0.0030	0.0156
Household size												
1 (ref)												
2—5	0.0436	−0.0441	0.0298	−0.1102	−0.1719	−0.0171	−0.2916	−0.0013	0.0049	0.0076	0.0008	0.0128
≥6	−0.3273	0.0049	−0.0556	0.1227	0.1756	0.0272	0.3819	−0.0003	0.0006	0.0009	0.0001	0.0019
Dietary knowledge score	0.0464	−0.1034	−0.0093	0.0254	−0.0763	0.0994	0.2184	0.0010	−0.0026	0.0079	−0.0103	−0.0226
Working status												
No (ref)												
Yes	−0.0053	1.6303	0.0077	0.0033	0.0253	−0.0133	−0.0093	0.0126	0.0053	0.0413	−0.0218	−0.0151
Urbanization index	0.0771	−0.1322	0.1335	0.2693	−0.3718	0.7755	1.6326	−0.0176	−0.0356	0.0491	−0.1025	−0.2157
Geographic regions												
Eastern (ref)												
Central	−0.0903	−0.1192	−0.0049	−0.0021	−0.0768	0.0779	0.0751	0.0006	0.0003	0.0092	−0.0093	−0.0089
Western	−0.2330	−0.2646	0.0842	0.0140	0.0476	0.0281	0.0556	−0.0223	−0.0037	−0.0126	−0.0074	−0.0147

Note: the sample size is 17,096.
